# Synthesis and Characterization of Novel Hybrid Flocculants Based on Potato Starch Copolymers with Hollow Carbon Spheres

**DOI:** 10.3390/ma14061498

**Published:** 2021-03-18

**Authors:** Beata Schmidt, Krzysztof Kowalczyk, Beata Zielinska

**Affiliations:** 1 Department of Chemical Organic Technology and Polymeric Materials, Faculty of Chemical Technology and Engineering, West Pomeranian University of Technology in Szczecin, 70-322 Szczecin, Poland; Krzysztof.Kowalczyk@zut.edu.pl; 2 Department of Nanomaterials Physicochemistry, Faculty of Chemical Technology and Engineering, West Pomeranian University of Technology in Szczecin, 70-322 Szczecin, Poland; Beata.Zielinska@zut.edu.pl

**Keywords:** flocculants, hybrid flocculants, potato starch, starch copolymers, polyacrylamide, mesoporous carbon spheres, starch-g-polyacrylamide

## Abstract

Novel carbon nanofiller-based starch-g-polyacrylamide hybrid flocculation materials (St-PAM-CS) were in situ prepared using potato starch (St), acrylamide (AM), and hollow mesoporous carbon spheres (CSs; diameters of 300–400 nm). Structures of different St-PAM-CS systems were characterized by Fourier transform infrared (FTIR) spectroscopy, X-Ray diffraction (XRD), differential scanning calorimetry (DSC), thermogravimetric analysis (TGA), laser scanning microscopy (LSM), and particle size analysis. The flocculation tests were evaluated by removing high turbidity kaolin suspension—initial absorbance 1.84. The effect of the St to AM molar ratio, doses, and content of CSs in hybrids on flocculation efficiency were examined. Satisfactory flocculation efficiency was obtained for all hybrids with 1 wt.% of the CS component. The highest reduction of the kaolin suspension absorbance (to 0.06) was observed for a 3 mL dose of the starch hybrid with the highest AM content. Additionally, St-PAM-CS showed a reduction in the sludge volume in time. The hybrids reached better flocculation efficiency in relation to the reference systems without CSs. The proposed flocculation mechanism (considering bridging, patching, and formation of hydrogen bonds) has been confirmed by the recorded results.

## 1. Introduction

Recently, due to continuous industrial, economic, and civilization development, one of the main problems is pollution of the natural environment (air, water, and soil). The growing world population significantly contributes to the pollution of water [1,2,3,4]. Therefore, control and reduction of water contamination is a crucial challenge. Various types of chemicals and water treatment agents are used for purifying water and sewage. Thus, coagulants and flocculants play the most important role in the field of waste water treatment [5,6,7,8]. However, simple coagulants and flocculating agents require high doses, which increase costs of the purification process. They must be dosed in the right order to keep their proper effectiveness. Conventional coagulants are based on inorganic particles whereas flocculants are organic polymeric compounds [5,6,7,8,9,10]. Unfortunately, during application of traditional waste water treatment agents the remaining of metal ions originating from inorganic coagulants and unreacted monomers from polymeric compounds are of major concern [11,12,13,14,15]. Therefore, a development of new water purification agents with comprehensive activity (e.g., dual functionality-flocculation and antibacterial action [16,17,18], coagulation and flocculation [19,20,21,22]), and/or containing natural polymers [23,24,25,26] or hybrid materials [27,28,29,30] is justified from a scientific, practical, and economic point of view.

Starch is a natural and renewable polymeric material, environmentally friendly, biodegradable and easily available. Due to its low solubility in water, low molecular weight and lack of charge properties, starch is most often found as a co-monomer of grafted flocculants [16,17,18,23,26,31] or in a form modified by e.g., etherification, esterification, or reactive extrusion processes [14,32,33,34]. Often used starch flocculants are copolymers of starch with polyacrylamide [23,31,33,34,35,36], which have the properties of both polymers: they are water-soluble due to the hydrophilic groups (–OH and –CONH_2_) presence in the chain and their water absorption increases with increasing molecular weight. Studies on hybrid flocculants based on polyacrylamide are rarely found in the literature [37,38,39], however, papers presenting hybrids of starch grafted polyacrylamide copolymers (additionally containing (nano)particles [28,29] or minerals, and carbon or inorganic forms) are more unique. The use of carbon spheres with sodium polystyrene sulfonate [40] or hollowed carbon spheres themselves as flocculants [41] are already reported in the literature.

In this work, novel hybrid flocculants based on potato starch polyacrylamide copolymers with hollow carbon spheres were successfully synthesized and characterized. The authors prepared and investigated the carbon spheres according to the procedure described in [42]. In the study reported here, for the first time, polyacrylamide was grafted onto starch in the presence of the mesoporous, hollow ball-shaped CSs with a diameter of 300–400 nm. Importantly, the starch-polyacrylamide-CS hybrids were obtained without surfactants, while maintaining the preparation regime such as sonication, mixing, and the order of adding ingredients. The procedures of the investigated nanocomposite starch- based flocculants with carbon spheres were described in the patent application form [43]. The materials were characterized by many methods: high transmission electron microscopy (TEM), laser scanning microscopy (LSM), X-ray diffraction analysis (XRD), Fourier transform infrared (FTIR) spectroscopy, thermogravimetric analysis (TGA), and differential scanning calorimetry (DSC). The article presents the effect of the dose, structure, composition, and grain size of the hybrid polymer system on the flocculation process in a model kaolin aqueous suspension in a standard jar test. The research revealed satisfactory flocculation efficiency of the new hybrid materials, however, no results for exact materials were found in the available literature. Moreover, the original hypothetical mechanisms of the flocculation activity of the starch-polyacrylamide-CS hybrids are presented.

## 2. Materials and Methods

### 2.1. Materials

Starch grafted polyacrylamide hybrids with carbon spheres were prepared using: (i) acrylamide (AM) (~99%, Merck, Darmstadt, Germany), (ii) potato starch (St, standard quality) with moisture content of ca. 16.5 wt.%, average molecular weight of 4.37 × 10^7^ g/mol, and 29.7 wt.% of amylose (Potato Industrial Enterprise “Nowamyl”, Nowogard, Poland), (iii) ammonium persulfate (ASP) as a polymerization initiator (~99%, Chempur, Piekary Slaskie, Poland), and (iv) acetone (analytical reagent grade) (Chempur). Kaolin (II Quality, Alfa Laval, Bratislava, Slovakia) was used as an inorganic pollutant in the jar tests. All materials were used without further purification.

The reagents used for the synthesis of the carbon spheres (purity of 99%) were supplied by the Sigma–Aldrich Chemie GmbH (Monachium, Germany).

### 2.2. Preparation of the Carbon Spheres

#### 2.2.1. SiO_2_@m-SiO_2__C18TMS Template Synthesis

The disordered mesoporous hollow silica spheres templates (SiO_2_@m-SiO_2__C18TMS) were produced using octadecyltrimethoxysilane (C18TMS; surfactant) and tetraethyl orthosilicate (TEOS; silica source). Firstly, 3.0 mL of TEOS was added to a solution containing 50 mL ethanol, 4.0 mL ammonia (28 wt.%), and 4.0 mL deionized water. The resulting mixture was stirred for 24 h. Afterward, C18TMS (1.0 mL) and TEOS (2.5 mL) mixture was added dropwise to the above-mentioned composition and it was alternately sonicated and stirred for 6 h. The obtained product was separated by filtration from the mixture, washed with ethanol and water, and finally dried at 100 °C for 24 h.

#### 2.2.2. Formation of the Carbon Spheres

The procedure of the mesoporous hollow carbon spheres preparation was following the method described by Wenelska et al. [42]. Briefly, the SiO_2_@m-SiO_2__C18TMS was situated in an alumina crucible in a tube furnace. The furnace was continuously purged by argon (100 sccm) and ethylene (30 sccm). A chemical vapor deposition (CVD) process was realized at 800 °C for 3 h. Next, the furnace was cooled to room temperature (the argon atmosphere). Afterward, the resulting SiO_2_@m-SiO_2__C sample was treated with hydrofluoric acid (HF) to remove the silica and the process was repeated twice. The obtained carbon spheres (CSs) were washed with distilled water (several times) and dried at 100 °C for 24 h.

### 2.3. Synthesis of Starch Grafted Polyacrylamide with Carbon Spheres

The starch grafted polyacrylamide copolymers (St-g-PAM) and appropriate hybrids St-g-PAM with carbon spheres (1.0 wt.% and 3.0 wt.% based on the weight of acrylamide) were prepared at the acrylamide molar concentration of 0.3, 0.5, or 0.7 mol/L and at constant concentration of starch (0.1 mol/L). The preparation procedure was similar to the method described in [28]. A dry starch dose was gelatinized in distilled water under mixing (200 rpm) in a glass rector (80 °C, 30 min) for 30 min. After gelatinization of starch, a sonicated aqueous suspension of CSs (amplitude of 20%, a cycle of 0.5, UP 200 ultrasonic homogenizer with the BS4 sonotode; Dr. Hielscher, Teltow, Germany) was introduced into the reactor under nitrogen atmosphere. The grafting reaction was realized at 42–46 °C for 3 h. The ASP was used in 1.0 wt.% on the weight of AM.

The product was washed several times with distilled water and methanol in order to remove an ungrafted polyacrylamide, and subsequently dried in a vacuum oven at 50 °C for 16 h.

### 2.4. Characterization Methods

High transmission electron microscopy (TEM) was used for examination of the structural details of the CS samples (Tecnai F30 with a field emission gun operating at 200 kV, FEI, Warrendale, PA, USA).

FTIR spectra of dried powdered samples of the St-g-PAM copolymers and the St-g-PAM-CS hybrids were recorded using the Nexus FTIR spectrometer (Thermo Nicolet Corp., Waltham, MA, USA) with the ATR Golden Gate accessory and the OMNIC software.

The powdered hybrid flocculants and CSs were specified by the XRD method. The materials were investigated using the Empyrean X-ray diffractometer (CuKα, λ = 1.544 Å, 7–90°, 0.01°/s; Malvern Panalytical, Malvern, UK). Microstructures of materials were characterized at a voltage of 40 kV and a tube current of 7.5 mA.

The surface morphology of selected freeze-dried hybrids (temperature < −50 °C, pressure < 0.120 mbar, time = 48 h; Christ Alpha 1–2 LD plus Instrument) was investigated using the laser scanning microscopy (LSM), (VK 9700, Keyence, Mechelen, Belgium) equipped with a violet laser source (wavelength of 408 nm) and a pin hole optical system. Surface images of the samples were done using the VK Analyzer software (Keyence, Mechelen, Belgium).

Thermogravimetric analysis (TGA) of the polymeric materials and the carbon spheres was executed using the TGA Q5000 thermal analyzer (TA Instruments, New Castle, DE, USA). The samples were heated from 20 to 900 °C with a heating rate of 20 °C/min (air atmosphere).

Differential scanning calorimetry analysis of the polymeric materials were realized by means of the DSC Q100 apparatus (TA Instruments, New Castle, DE, USA). Samples (~5 mg) were tested using hermetic aluminum pans at the temperature range of 0–350 °C (with a heating rate of 20 °C/min).

The particle size analysis of CS-based polymer hybrids was realized using the dynamic laser light scattering method (Zetasizer Nano ZS; Malvern Instruments, Malvern, UK). Scans were collected 3 times every 2 min.

The UV-Vis spectrophotometer (UV-9000; BioSens, Ningbo, China) was used for characterization of a flocculation process of a model kaolin suspension (5 g of kaolin in 1 L of distilled water). The appropriate amount of a tested material was added to a beaker under mixing (with decreasing speed of mixing from 500 to 50 rpm within 10 min). Then, the entire suspension was immediately transported to a calibrated Imhoff funnel. Absorbance of the solution above the sediment was measured at the wavelength 470 nm for 2 h (with intervals of 10 min). Flocculation properties of the hybrid nanocomposites were compared to the St-g-PAM reference systems and untreated kaolin suspension. Additionally, a volume of the settled deposit was noted. Each experiment was performed three times and the results were averaged. Relative error values were lower than 3.0%. The pH of the model kaolin suspension was 7.2.

## 3. Results and Discussion

### 3.1. Properties of the Prepared Carbon Sphere

**Figure 1:**
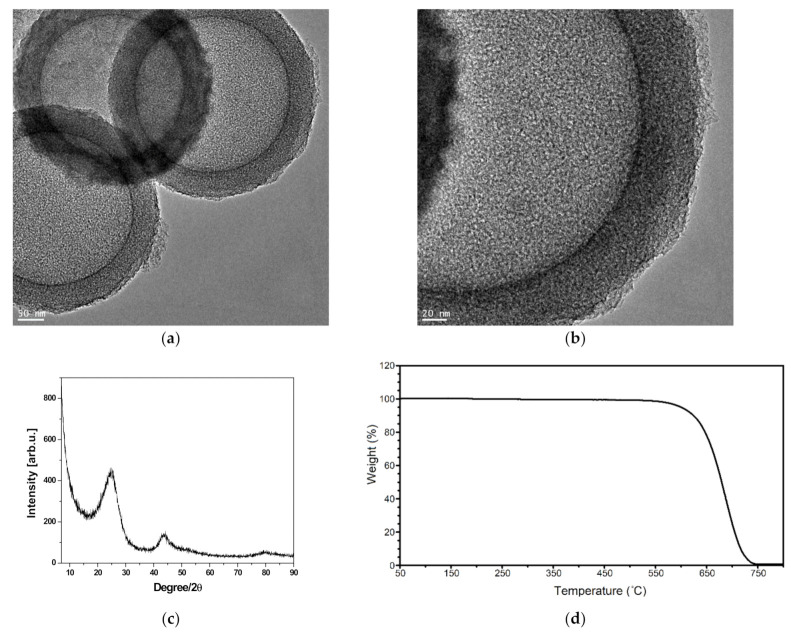
Characterization of carbon spheres: (**a**) transmission electron microscopy (TEM) pictures of a carbon spheres group; (**b**) TEM pictures of single carbon sphere; (**c**) a wide angle X-ray powder diffraction spectrum; and (**d**) thermogravimetric curve.

TEM images as well as an XRD spectrum and a TGA curve for the prepared carbon spheres are shown in Figure 1. As presented in Figure 1a,b, the hollow CSs with diameters of 360–420 nm and the disarranged mesoporous shell thickness of ca. 50 nm can be expressly observed. The XRD pattern of the CSs exhibit two wide diffraction peaks at 25° and 45° 2θ adequate to the (002) and (100) planes of the graphitic carbon, respectively [44]. TGA results demonstrated that burning of the raw CSs begins at ca. 580 °C. Then the weight loss swiftly rises until the total of CSs disappear at about 750 °C. The residual weight of the carbon spheres after the heating process can be practically neglected, suggesting a high purity of the synthesized CSs.

### 3.2. Starch Grafted Polyacrylamide with Carbon Spheres

A series of starch grafted polyacrylamide copolymers (St-PAM) and the corresponding carbon spheres-based hybrids (St-PAM-CS) with different AM and CS content were successfully synthesized. Details of the polymerization process such as molar ratio of the substrates, yield of the reaction as well as symbols of samples are given in Table 1. Generally, the concentration of potato starch was always 0.1 mol/L; the reaction yield ranged from 82% to 88% and it was slightly higher for the polymerization processes realized without the CS addition.

**Scheme 1:**
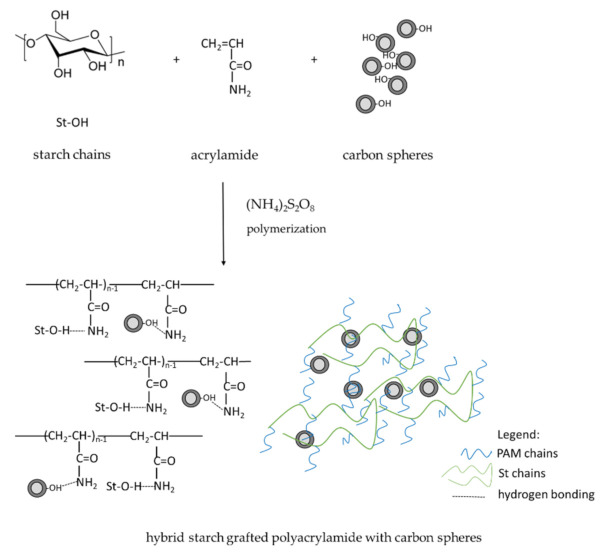
Schematic illustration of starch grafting polyacrylamide reaction with visualization of hybrid formation.

The modification of starch was performed by free radical polymerization of potato St with AM in the presence of CSs. Scheme 1 presents the starch grafting polyacrylamide reaction with visualization of hybrid formation. The starch has three –OH groups on the repeating unit and most starch backbones can be used for free radical grafting. Monomer (AM) reacts with the activated starch to form side chains attached via strong hydrogen bonding. Additionally, the presence of the –OH and C=O groups on the carbon spheres was confirmed, which may improve the hydrophilicity of CSs in water systems [45,46]. Therefore, during the reaction, the –OH groups can be activated and hydrogen bonded with PAM. As a result, the mesoporous carbon spheres stabilized by hydrogen bonds, embedded, penetrated, or entwined by polyacrylamide chains (grafted onto starch chains) were obtained.

### 3.3. Properties of Starch Grafted Polyacrylamide with CS Hybrids

#### 3.3.1. Chemical Structure

**Figure 2:**
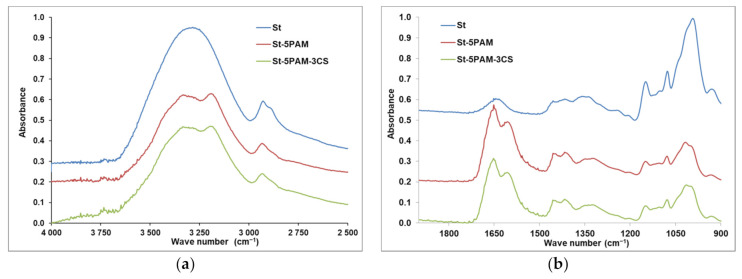
Fourier transform infrared (FTIR) spectra of native potato starch (St), starch grafted polyacrylamide copolymer with the St:AM molar ratio of 1:5 (St-5PAM) and starch grafted polyacrylamide hybrid with the St:AM molar ratio of 1:5 and 3 wt.% of CSs (St-5PAM-3CS) in characteristic wavenumber ranges: (**a**) 4000–2500 cm^−1^ and (**b**) 1900–900 cm^−1^.

The proof for the chemical modification of starch and creation of the polymer hybrids was supplied by FTIR test results. Figure 2 shows the FTIR spectra for native St, starch grafted polyacrylamide copolymer with St:AM molar ratio of 1:5 (St-5PAM) and for the relevant system containing 3 wt.% of CSs (St-5PAM-3CS) (in the wavenumber ranges of 4000–2500 cm^−1^ in Figure 2a and 1900–900 cm^−1^ in Figure 2b). In a typical FTIR spectrum of starch, the absorption band at the wavelength range of 3650–3000 cm^−1^ is ascribed to the –OH groups stretching vibrations while the band at 2900 cm^−1^ represents C–H and symmetrical CH_2_ groups stretching vibrations. The absorption bands at 1160, 1080, and 1015 cm^−1^, caused by C–O–C stretching vibrations are also characteristic for starch. Moreover, the absorption bands at 1460 and 1380 cm^−1^ correspond to O–H in-plane bending vibrations. The peak at 1640 cm^−1^ can be assigned to the water absorbed by the starch. (Figure 2b) [47,48].

The confirmation of PAM grafting onto starch (St-5PAM and St-5PAM-3CS) are two absorption bands (in the wavelength range of 3650–3000 cm^−1^) derived from –OH groups and –N–H groups of PAM (Figure 2a) and three characteristic groups resulting from CONH_2_, i.e.,: –C=O at 1680 cm^−1^, –N–H at 1610 cm^−1^, and –C–N at 1405 cm^−1^ (Figure 2b) [49]. The FTIR spectra of the CS-based hybrids (St-5PAM-3CS) are identical to those recorded for the copolymers (St-5PAM) since the carbon spheres exhibit similar absorption peaks (the –OH and –C=O groups; Supplemental Figure S1)—the spectra coincide.

#### 3.3.2. Crystallinity

**Figure 3:**
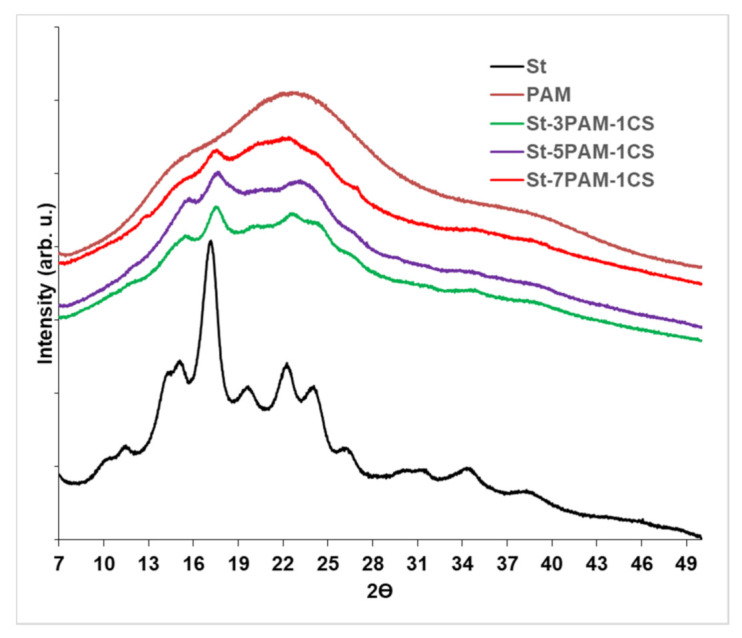
Wide angle X-ray powder diffraction spectra for starch (St), polyacrylamide (PAM), and starch grafted polyacrylamide hybrids with 1 wt.% of CSs and the St:AM molar ratio of: 1:3 (St-3PAM-1CS), 1:5 (St-5PAM-1CS), and 1:7 (St-7PAM-1CS).

Crystallographic characteristics of the prepared samples were examined by the XRD method. The wide angle XRD spectra for St, PAM, and starch grafted polyacrylamide hybrids with 1 wt.% of CSs and the St:AM molar ratio of: 1:3 (St-3PAM-1CS), 1:5 (St-5PAM-1CS), and 1:7 (St-7PAM-1CS) were shown in Figure 3. The starch indicates a considerable diffraction peak at ca. 17.0° 2θ, three weak peaks at ca. 20°, 22°, and 25° 2θ and, an extra peak at 15° 2θ (characteristic scattering features of B-type crystal from the retrograded starch chains [49]). The PAM exhibited only one broad diffraction peak at ca. 23.3° 2θ (the maximum) indicating almost amorphous nature of the polymers [48,49].

The XRD results clearly shown changes in composition of the obtained hybrids. In the spectra for the hybrids, the narrow starch crystal peaks disappear and a wide halo appears from PAM. The type of the XRD pattern changes from crystalline to semi-crystalline (or to nearly amorphous structure) with increasing PAM content in the hybrid copolymers. This represents destruction of the starch crystal structure during the grafting reactions. All the hybrids samples (from St-3PAM-1CS to St-7PAM-1CS) exhibit a strongly reduced peak at 17.0° 2θ (characteristic for starch) and a weak peak around 25.0° 2θ (characteristic for the carbon spheres). The XRD curves for the polymer composites indicated that the CSs are located in the hybrid polymer matrix. The XRD spectra for samples containing the highest CS content (3 wt.% of CSs) are comparable with these recorded for St-PAM-1CS (Figure 3).

#### 3.3.3. Thermal Features

**Figure 4:**
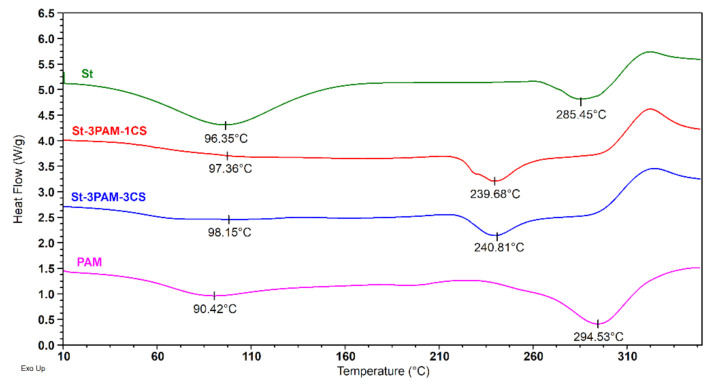
Differential scanning calorimetry (DSC) thermographs for native potato starch (St), polyacrylamide (PAM), and starch grafted polyacrylamide hybrids with 1 wt.% of CSs (St-3PAM-1CS) or 3 wt.% of CSs (St-3PAM-3CS) and the St:AM molar ratio of 1:3.

DSC curves for St, PAM, and the representative hybrids containing 1 wt.% of CSs (St-3PAM-1CS) and 3 wt.% of CSs (St-3PAM-3CS) are presented in Figure 4. The starch is a semi-crystalline material with a gelatinization temperature peak (T_p_) at 96.35 °C and melting temperature of crystallites (T_m_) at 285 °C. The observed T_m_ is higher than the value reported by Díaz and others [50], but the variance may be due to differences in the starch source and heating rate. DSC studies of PAM show two endothermic peaks. The first was observed at 60–110 °C—it corresponds to evaporation of water absorbed by the material. The second peak (294 °C) represents decomposition of PAM amide groups and generation/evaporation of ammonia [51]. Broad endothermic peaks at 220–250 °C (recorded for the hybrids) are assigned to the melting processes (T_m_) due to the semi-crystalline structure of the starting substances. The depth of the T_m_ peak demonstrates reduction of the crystallinity degree of materials and an increment of amorphous fraction content. Generally, the observations correlate with these noted for the XRD data. On the other hand, the DSC results correspond with the TGA results up to the temperature of 300 °C [52].

**Figure 5:**
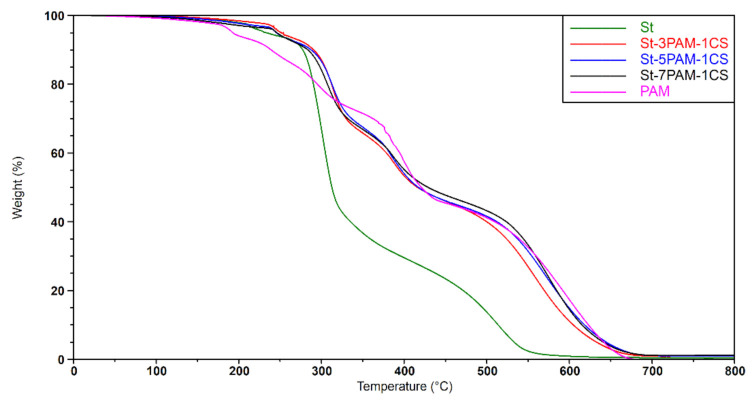
Thermogravimetric analysis (TGA) thermographs for potato starch (St), polyacrylamide (PAM), and starch grafted polyacrylamide hybrids with 1 wt.% of CSs and different St:AM molar ratio: 1:3 (St-3PAM-1CS), 1:5 (St-5PAM-1CS), and 1:7 (St-7PAM-1CS).

Figure 5 presents the TGA curves for St, PAM, and the polymer hybrids with different PAM and CS content (composites with 1 and 3 wt.% of CSs show similar tendencies during their heating). As can be seen, starch exhibit relatively significant and the fastest weight loss at the range of 300–320 °C. The weight loss of PAM is almost uniform up to 400 °C, which is attributed to the major thermal decomposition of functional groups in polymer chains and generation of gases, such as CO, OH, and NH_2_ [52]. The final thermal decomposition of PAM backbone occurred above 400 °C. All of the TGA curves for the hybrids are smooth with nearly similar behaviors during the test. These thermographs are similar to the TGA curve of starch heated in the temperature range of 10–300 °C and to the TGA curve of PAM above this temperature. The polymer hybrids are characterized by the following regions of weight loss: dehydration at 30–220 °C, degradation of starch, and decomposition functional groups at 220–400 °C, and degradation of PAM chains at 400–650 °C. This trend of the hybrids thermal curves shows that the nanocomposites are more thermally stable in relation to starch, even with a high St content in the hybrid materials.

#### 3.3.4. Surface Morphology

**Figure 6:**
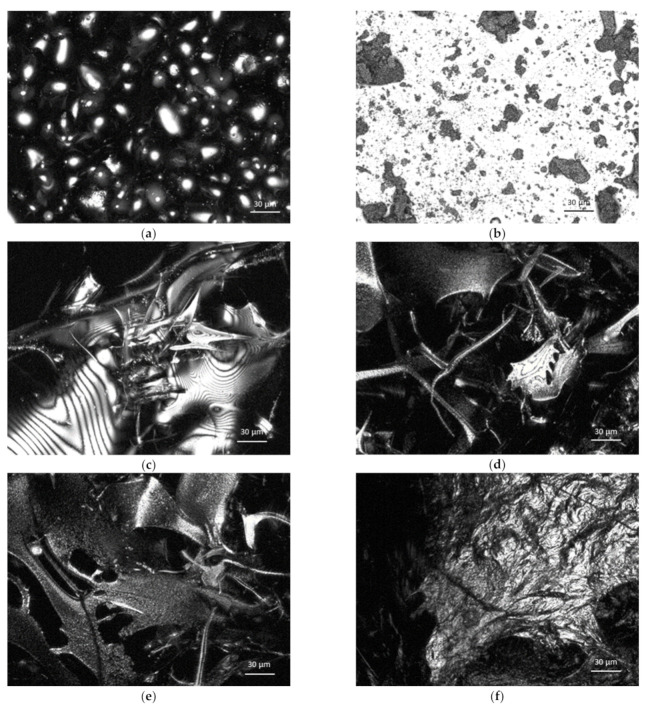
Laser scanning microscopy (LSM) images of (**a**) native potato starch; (**b**) carbon spheres aggregates; (**c**) PAM; (**d**) starch grafted polyacrylamide hybrid with the St:AM molar ratio of 1:3 and 1 wt.% of CSs (St-3PAM-1CS); (**e**) polymer hybrid with the St:AM molar ratio of 1:5 and 1 wt.% of CSs (St-5PAM-1CS); and (**f**) polymer hybrid with the St:AM molar ratio of 1:3 and 3 wt.% of CSs (St-3PAM-3CS).

LSM micrographs (magnification of 1.000×) for pure starch, carbon spheres, polyacrylamide, and selected polymer hybrids are shown in Figure 6. LSM images for selected materials with different magnification are presented in Figure S2 in the supplementary data. Potato starch occurs in the form of nonuniform, spherical granules with various sizes (Figure 6a) while the carbon spheres appear at clusters with different dimensions (Figure 6b). The PAM is a transparent and smooth material in the form of jagged and bent lamellas (Figure 6c). Surfaces of the CS-based polymer hybrids (Figure 6d–f) are generally rough due to the carbon spheres’ presence. The LSM of the CS composites revealed more heterogeneous surfaces compared to polyacrylamide structure. The LSM method is a suitable technique for visualizing differences in surface morphology between the polymers and the prepared hybrids.

#### 3.3.5. Particle Size Analysis of Polymer Hybrids

**Figure 7:**
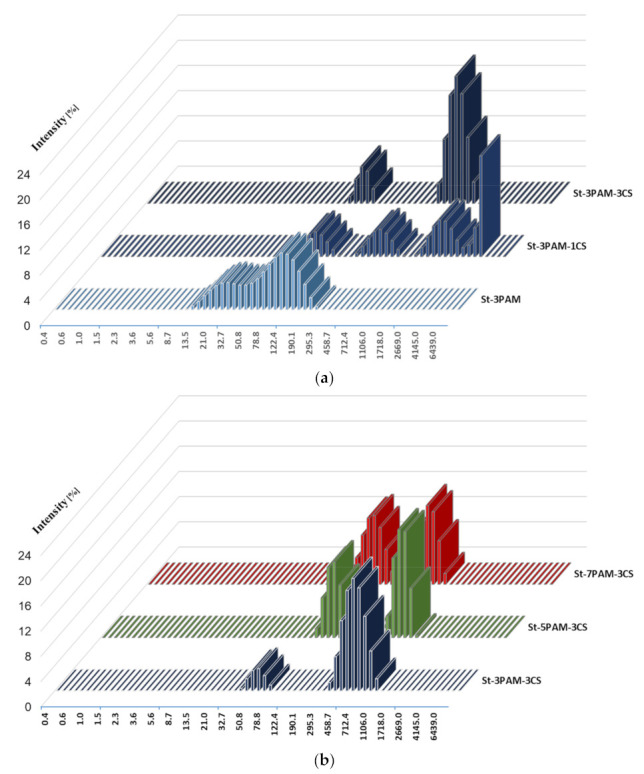
Particles size distributions for: (**a**) starch grafted polyacrylamide without CSs and the St:AM molar ratio of 1:3 (St-3PAM), hybrids with 1 wt.% of CSs (St-3PAM-1CS) and 3 wt.% of CSs (St-3PAM-3CS); (**b**) hybrids with constant 3 wt.% of CSs and different St:AM molar ratio: 1:3 (St-3PAM-3CS), 1:5 (St-5PAM-3CS), and 1:7 (St-7PAM-3CS).

The particles size distribution (PSD) was investigated using the Zetasizer Nano ZS apparatus in an aqueous environment; PSD for the materials with the constant St:AM molar ratio (1:3) is presented in Figure 7a while PSD for the hybrids with 3 wt.% of CSs and different St:AM molar ratio is shown in Figure 7b. In detail, these figures presents both the size of polymers and polymer hybrids as well as content of selected particles in the materials. Generally, the starch grafted polyacrylamide copolymer (St-3PAM) may be considered as relatively homogeneous material with a particles size in the range of 10–300 nm [53]. In contrast, the CS hybrids do not exhibit similar particles size uniformity. In all discussed groups of materials, the hybrids containing 1 wt.% of CSs have the largest particle sizes (100–4800 nm; St-3PAM-1CS). However, the division into particles size fractions suggests that not all the molecules of copolymer were modified by CSs, which is possible due to such a low nanofiller content. The PSD values for the materials with 3 wt.% of CSs clearly indicate formation of the polymer hybrids-particle sizes ranging from 300 to 1100 nm (Figure 7b). It should be noted that increasing content of PAM in the hybrid causes a slight shift of the particles size values to smaller diameters: 350–1100 nm for St-3PAM-3CS, 450–1000 nm for St-5PAM-3CS, and 300–700 nm for St-7PAM-3CS.

### 3.4. Flocculation Effectiveness

#### 3.4.1. Jar Tests

**Figure 8:**
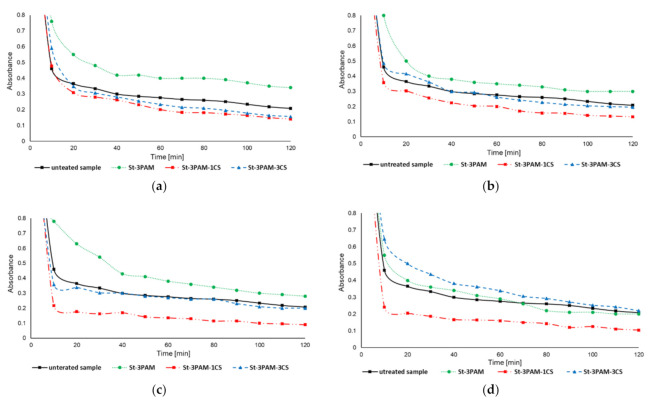
Absorbance for kaolin aqueous suspension as a function of time for starch grafted polyacrylamide with the St:AM molar ratio of 1:3 (St-3PAM), and its hybrids with 1 wt.% of CSs (St-3PAM-1CS) and 3 wt.% of CSs (St-3PAM-3CS) in doses: (**a**) 1 mL; (**b**) 3 mL; (**c**) 5 mL; and (**d**) 10 mL.

**Figure 9:**
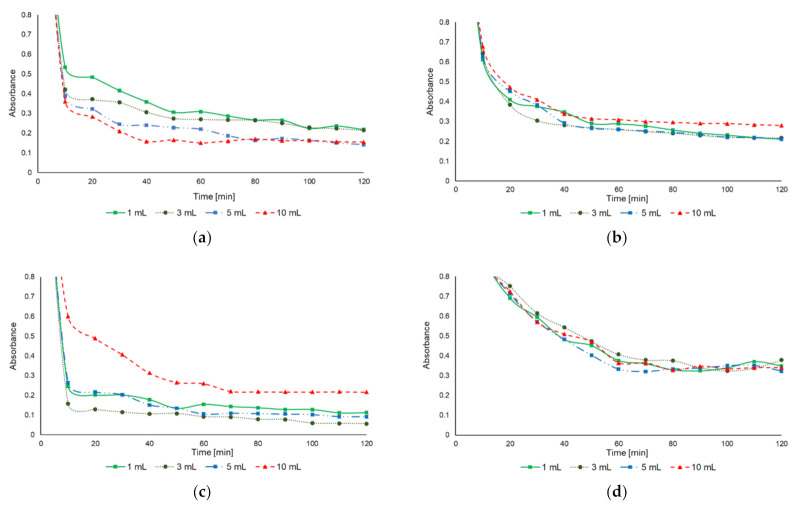
Absorbance for kaolin aqueous suspension as a function of time at different doses of: (**a**) St-5PAM-1CS; (**b**) St-5PAM-3CS; (**c**) St-7PAM-1CS; or (**d**) St-7PAM-3CS.

Flocculation efficiency of starch grafted polyacrylamide copolymers and their hybrids, as a dependence of absorbance as a function of time for different doses of flocculants, was investigated by means of a model kaolin suspension. The flocculation study of the polymeric materials with the St:AM molar ratio of 1:3 (and with the different CS content) was graphically illustrated in Figure 8. Additionally, the jar test results for the prepared polymeric flocculants with the St:AM molar ratio of 1:5 or 1:7 are presented in Figure 9. Generally, the various doses (i.e., 1, 3, 5, and 10 mL) of the flocculants were investigated. They correspond to the polymer concentration of 1, 3, 5, and 10 ppm, respectively. The initial absorbance for the kaolin suspension was 1.84.

In this case, it is clearly indicated that the reference copolymer and its hybrid (with the higher CS content) do not exhibit flocculation properties. On the other hand, the St-3PAM-1CS hybrid material (1 wt.% of CSs) has flocculation ability at all doses used (the final reduction of kaolin suspension turbidity was in the range of 0.09–0.13. The best results were noted for the 5mL dose) due to the polymer network formed and the additional load in consequence of the presence of CS in material. Generally, the recorded flocculation test results correlate with the aforementioned PSD parameter values, i.e., St-3PAM-1CS has the largest particles, which indicate a higher hydrodynamic volume, hydrodynamic radius, and a higher weight in contrast to the St-3PAM and St-3PAM-3CS particles.

Similar flocculation characteristics to the group of hybrids with 3PAM are also exhibited by hybrids with a molar ratio of St-AM equal to 1:5 and 1:7—Figure 9. Only hybrids containing 1 wt.% of CSs present kaolin flocculation ability (Figure 9a,c). As can be seen in Figure 9a, only 3 mL and 5 mL flocculant doses of St-5PAM-1CS causes a slight improvement in the transparency of the suspension. In contrary, the hybrid St-7PAM-1CS, due to the high content of polyacrylamide in its composition, exhibits noticeable flocculating properties for all doses except the highest one—10 mL (Figure 9c). In the case of an excess of polymer and high CS content, there is no longer enough exposed particle surfaces to attach the segments and the particles may repel each other or the presence of CS blocks expansion of the polymer chains (Figure 9b,d). The best results in the absorbance reduction of kaolin suspension were obtained for 3 mL dose of starch hybrid with the highest content of PAM and 1 wt.% of CSs (St-7PAM-1CS): absorbance from 0.15 after 10 min jar test to 0.06 after 100 min—Figure 9c. For comparison, the absorbance for the untreated kaolin suspension were 0.46 and 0.21, respectively.

**Figure 10:**
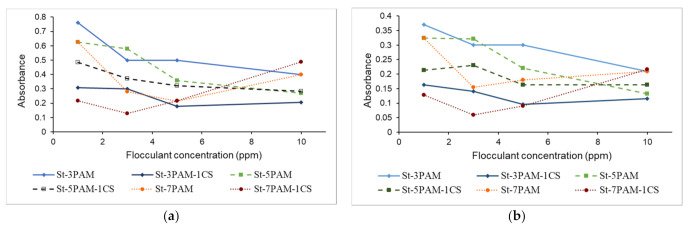
Absorbance for kaolin aqueous suspension as a function of concentration of starch grafted polyacrylamide with different PAM content and their hybrids with 1 wt.% of CSs after: (**a**) 20 min and (**b**) 100 min of flocculation tests duration.

Figure 10 shows the absorbance changes of starch-g-polyacrylamide with different PAM content and their hybrids with 1 wt.% of CSs after 20 min (Figure 10a) and 100 min (Figure 10b) running flocculation test in all tested concentrations. It was noted that, according to the rules of flocculation, with an increase in flocculant concentration, more particles bind together to create larger flocs, and hence the turbidity of the suspension decreased. Contrariwise, above the ideal flocculant concentration, the turbidity of the kaolin suspension again increased as a result of the steric stabilization and electrostatic repulsion. The recapitulation in Figure 10 clearly shows that each hybrid exhibits better flocculation properties than its corresponding starch copolymer with PAM. It has been proven that, with regard to the hybrids, because of the branching nature as well as the existence of grafted PAM chains and the entity of the polymer network with carbon spheres, the nanocomposite materials are able to create stronger bridging with kaolin particles to form bigger flocs than the corresponding pure copolymer. Compared to the flocculating properties of fly ash or cationic polyacrylamide [41], carbon spheres with sodium polystyrene sulfonate [40], chitosan graft poly(acrylamide and methacryloyl ethyl trimethyl ammonium chloride) [24], polymer grafted starches [26], the use of starch grafted polyacrylamide with CS hybrids as flocculants for the kaolin suspension significantly accelerates the sedimentation process (about 10 min) and reduces turbidity at very low doses (3 ppm).

#### 3.4.2. Sludge Volume

**Figure 11:**
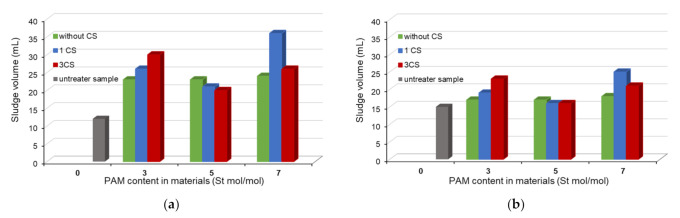
Sludge volume changes during the flocculation tests for a kaolin aqueous suspension dependent on the flocculants in 3 mL dose after: (**a**) 20 min and (**b**) 100 min of the test duration.

Figure 11 shows sludge volumes changes during the jar tests in relation to the material type and time (i.e., after 20 min and 100 min; 3 mL doses of the flocculants). Additionally, results for the untreated kaolin suspension were presented. As can be seen, the sludge volume for the untreated kaolin suspension increased from 12 mL (after 20 min) to 15 mL after 100 min of the test duration. In all cases, incorporation of the flocculants into the kaolin suspension caused an increment of the sediment volume (in relation to the reference system). Nevertheless, the prepared flocculants reduced the sludge volume (and the turbidity) of the kaolin suspension with extended duration of the test. It indicates compression of the material/sludge under the water pressure and/or a water removing process from the sludge. These compressible flocs may result from the hydrogen bonding between the nonionic polymer and the kaolin [41]. The highest sludge volume, 36 mL after 20 min, was reduced to 25 mL after 100 min (St-7PAM-1CS). This feature is highly desirable for application reasons, because a lower sludge volume means less discharged sludge from a sewage treatment plant.

#### 3.4.3. Flocculation Mechanism

**Scheme 2:**
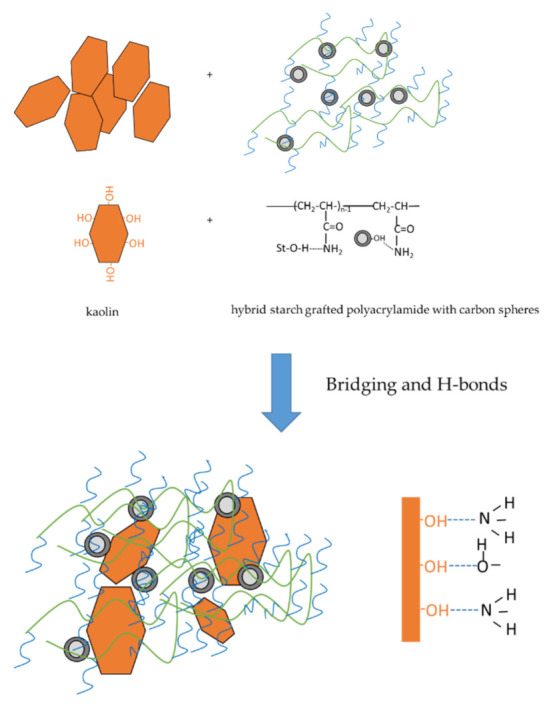
A scheme of possible flocculation mechanism for a CS-based starch grafted polyacrylamide/kaolin system.

Considering the influence of the CS-based starch grafted polyacrylamide hybrids type and dosage on flocculation efficiency and sludge volume as well as based on the literature reports [41,54,55] the possible flocculation mechanism was proposed (Scheme 2).

In the proposed mechanism of flocculation, long chain polymers with carbon spheres adsorb onto kaolin particles in such a manner that an individual chain can be attached to two or more particles, thus “bridging” them together. The created fine aggregates are connected and cross-linked together by patching and bridging during creation of hydrogen bonds between –OH groups of kaolin and –NH_2_ groups of PAM and –OH groups of St. The stability of CS in starch grafted polyacrylamide network is due to the hydrogen bonds between the hydroxyl groups on the CS and the amide groups on the grafted PAM. The presence of the carbon spheres in the polymeric material may cause an increment of the flocculants aggregates/agglomerates size and acceleration of flocs sedimentation. Lastly, the flocs are also generated by enmeshment and sweeping effect in the flocculation process.

## 4. Conclusions

Starch-grafted polyacrylamide hybrids (with different content of starch (St), polyacrylamide (PAM), and mesoporous carbon spheres (CSs)) were successfully obtained and characterized. The hybrids were synthesized via a free radical polymerization process preceded by preparation of a CS aqueous suspension. The CS-based polymeric hybrids were compared to the corresponding polymers without the nanofiller using Fourier transform infrared spectroscopy, X-ray diffraction analysis, as well as laser scanning microscopy. Additionally, differences between the materials were monitored via thermogravimetric analysis. Particles size analysis results revealed significantly larger size of the hybrid particles (100–4800 nm) in relation to the unfilled starch copolymer (10–300 nm). It was found that the hybrids with lower CS content (1 wt.%) exhibit excellent flocculation properties for a kaolin aqueous suspension. It should be noted that the flocculation properties strongly depend on the composition of the hybrid (mainly PAM and CS content) as well as on an applied flocculant dose. Generally, the best results in the absorbance reduction of the kaolin suspension (from 1.84 to 0.06) were noted for a 3 mL dose of the hybrid with the highest PAM content (St-7PAM-1CS). The very good results for a sludge volume measurement test were registered for this system as well. A volumetric reduction of a kaolin sludge (as a function of test duration) increases application possibilities of the prepared materials. The CS-based starch-grafted polyacrylamide hybrids showed better flocculation efficiency compared to their analogous without the CS nanofiller.

## Figures and Tables

**Table 1:** Detail of hybrids and starch grafted copolymer synthesis.

	Molar Ratio St:AM	Label of Samples		Mass of CSs (g)		Yield (%)	
Concentration of CSs (wt.%)		1	3	1	3	1	3
Hybrid-Starch Grafted Polyacrylamide with CSs	1:03	St-3PAM-1CS	St-3PAM-3CS	0.032	0.096	84	83
	1:05	St-5PAM-1CS	St-5PAM-3CS	0.053	0.159	85	86
	1:07	St-7PAM-1CS	St-7PAM-3CS	0.075	0.225	82	84
Copolymer- Starch Grafted Polyacrylamide	1:03	St-3PAM		0		88	
	1:05	St-5PAM		0		87	
	1:07	St-7PAM		0		85	

## Data Availability

Data is contained within the article or supplementary material.

## Supplementary Materials

The following are available online at https://www.mdpi.com/1996-1944/14/6/1498/s1, Figure S1: FTIR spectra of carbon spheres (CS); Figure S2: LSM images with different magnification: (**a**) and (**b**) native potato starch; (**c**) and (**d**) PAM; (**e**) and (**f**) starch grafted polyacrylamide hybrid with the St:AM molar ratio of 1:3 and 1 wt.% of CS (St-3PAM-1CS); (**g**) and (**h**) polymer hybrid with the St:AM molar ratio of 1:5 and 1 wt.% of CS (St-5PAM-1CS); (**i**) and (**j**) polymer hybrid with the St:AM molar ratio of 1:3 and 3 wt.% of CS (St-3PAM-3CS).
