# Supplementary material for: Synthesis and Characterization of Novel Hybrid Flocculants Based on Potato Starch Copolymers with Hollow Carbon Spheres
Source: Materials (Basel). 2021 Mar 18;14(6):1498. doi: 10.3390/ma14061498 (PMC8003131; doi:10.3390/ma14061498)
Supplement: Supplementary file 1 [file materials-14-01498-s001.pdf]

materials

MDPI

Article

Synthesis and Characterization of Novel Hybrid Flocculants Based on Potato Starch Copolymers with Hollow Carbon Spheres

Beata Schmidt [1,*], Krzysztof Kowalczyk [1] and Beata Zielinska [2]

[1] West Pomeranian University of Technology in Szczecin, Faculty of Chemical Technology and Engineering, Department of Chemical Organic Technology and Polymeric Materials, 70-322 Szczecin, Poland; Krzysztof.Kowalczyk@zut.edu.pl
[2] West Pomeranian University of Technology in Szczecin, Faculty of Chemical Technology and Engineering, Department of Nanomaterials Physicochemistry; Beata.Zielinska@zut.edu.pl
* Correspondence: Beata.Schmidt@zut.edu.pl; Tel.: +48914494749

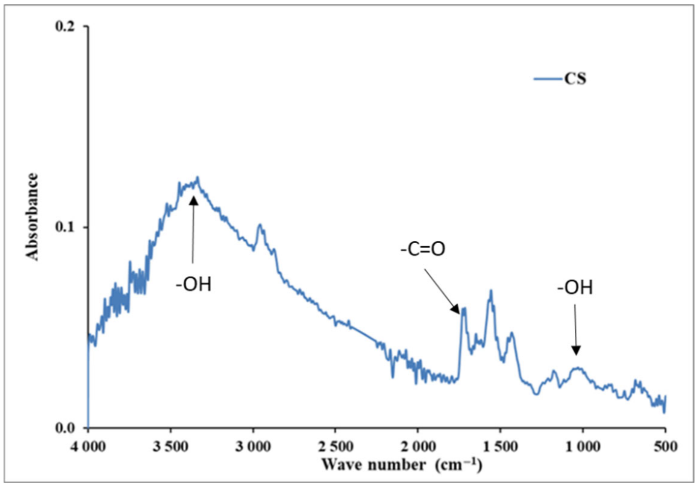

Figure 1. S. FTIR spectra of carbon spheres (CS).

Citation: Schmidt, B.; Kowalczyk, K.; Zielinska, B. Synthesis and characteristics of novel hybrid flocculants based on potato starch copolymers with hollow carbon spheres. *Materials* **2021**, *14*, 1498. https://doi.org/10.3390/ma14061498

Academic Editor: Ecaterina Matei

Received: 17 February 2021
Accepted: 16 March 2021
Published: 18 March 2021

Publisher's Note: MDPI stays neutral with regard to jurisdictional claims in published maps and institutional affiliations.

Copyright: © 2021 by the authors. Licensee MDPI, Basel, Switzerland. This article is an open access article distributed under the terms and conditions of the Creative Commons Attribution (CC BY) license (http://creativecommons.org/licenses/by/4.0/).

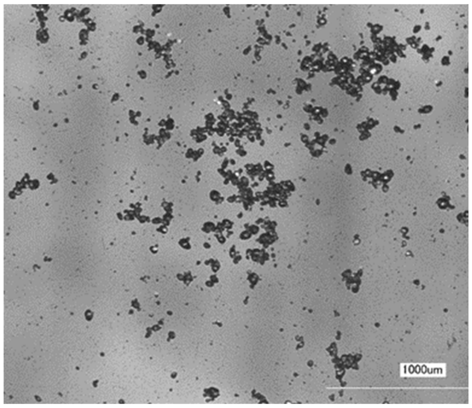

(**a**)

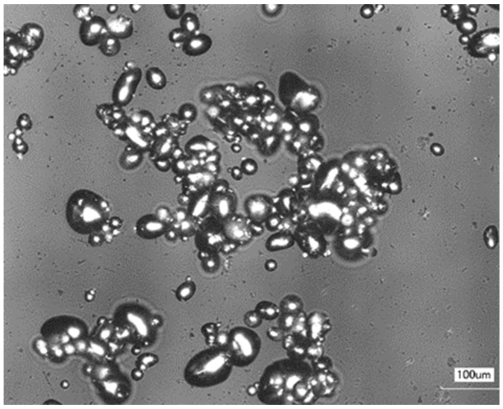

(**b**)

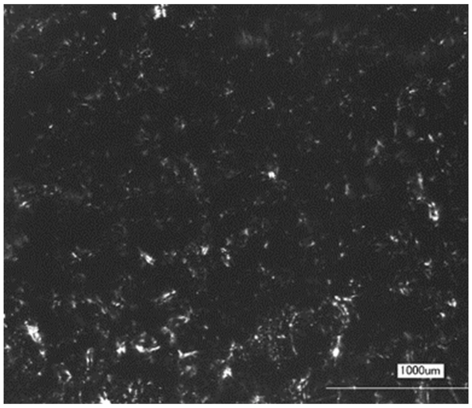

(**c**)

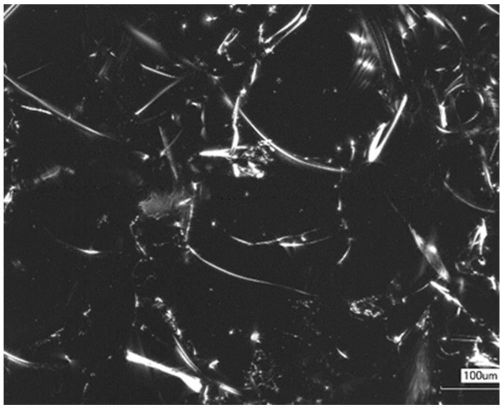

(**d**)

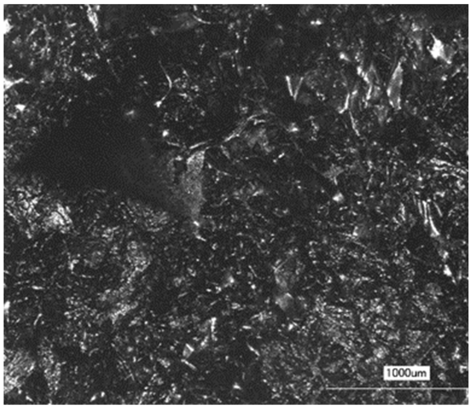

(**e**)

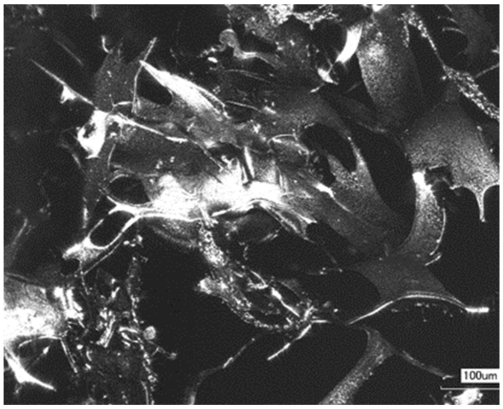

(**f**)

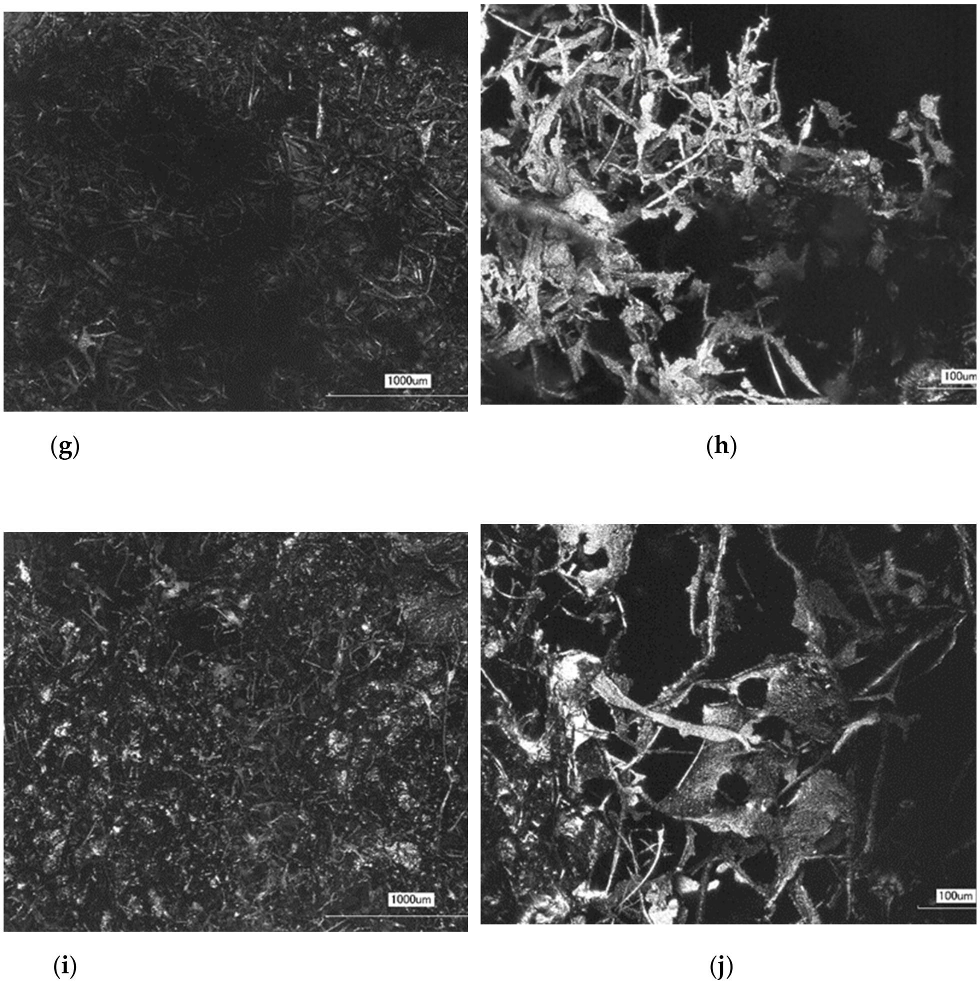

Figure 2. S. LSM images with different magnification: (**a**) and (**b**) native potato starch; (**c**) and (**d**) PAM; (**e**) and (**f**) starch grafted polyacrylamide hybrid with the St:AM molar ratio of 1:3 and 1 wt.% of CS (St-3PAM-1CS); (**g**) and (**h**) polymer hybrid with the St:AM molar ratio of 1:5 and 1 wt.% of CS (St-5PAM-1CS); (**i**) and (**j**) polymer hybrid with the St:AM molar ratio of 1:3 and 3 wt.% of CS (St-3PAM-3CS).
